# Supplementary material for: Increased aerosol transmission for B.1.1.7 (alpha variant) over lineage A variant of SARS-CoV-2
Source: bioRxiv. 2021 Jul 26:2021.07.26.453518. Preprint. [Version 1] doi: 10.1101/2021.07.26.453518 (PMC8328059; doi:10.1101/2021.07.26.453518)
Supplement: 1 [file NIHPP2021.07.26.453518V1-supplement-1.pdf]

bioRxiv preprint doi: https://doi.org/10.1101/2021.07.26.453518; this version posted July 26, 2021. The copyright holder for this preprint (which was not certified by peer review) is the author/funder. This article is a US Government work. It is not subject to copyright under 17 USC 105 and is also made available for use under a CC0 license.

Supplementary Material

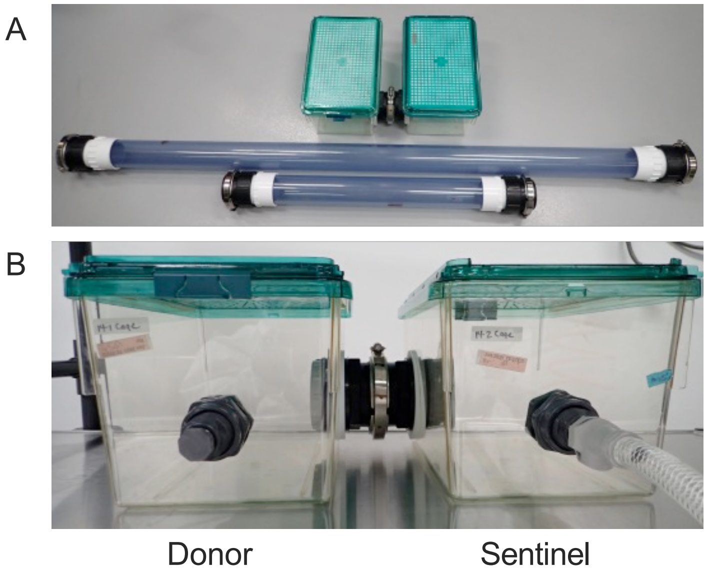

Supplemental Figure 1: Aerosol transmission cages. A/B. Design of a new caging system in which two hamster cages could be separated at 3 different distances. The distances chosen were nominally 16.5 cm, 106 cm and 200 cm. The distance could be adapted by swapping out a 76 mm inside diameter connection tube. Cages were installed on autoclavable stainless steel shelves (Metro) inside a BSL-4 containment laboratory. Airflow was measured by flowmeters mounted to the shelves. Air was pulled through the system by a negative-pressure pump (Vacuubrand) and filtered through a hepa-filter before the exhaust.

bioRxiv preprint doi: https://doi.org/10.1101/2021.07.26.453518; this version posted July 26, 2021. The copyright holder for this preprint (which was not certified by peer review) is the author/funder. This article is a US Government work. It is not subject to copyright under 17 USC 105 and is also made available for use under a CC0 license.

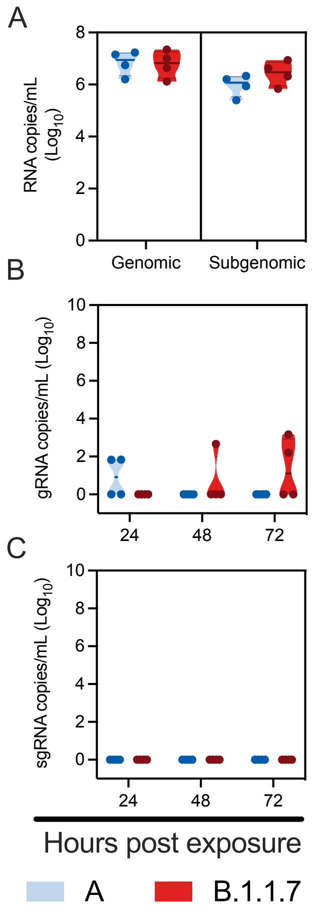

Supplementary Figure 2: B.1.1.7 and lineage A aerosol transmission efficiency at three days post inoculation. Comparison of aerosol transmission efficiency of lineage A and B.1.1.7 SARS-CoV-2 variants in the Syrian hamster. Donor Syrian hamsters were inoculated with 8x10[4] $TCID_{50}$ SARS-CoV-2 lineage A or B.1.1.7 variant. After 72 hours, donors were introduced to the upstream cage and sentinels (2:2 ratio) into the downstream cage. Exposure was limited to one hour for B.1.1.7 and lineage A (N = 4, respectively). **A.** Viral load in oropharyngeal swabs of donors collected 72 hours post inoculation was measured by gRNA and sgRNA. **B/C.** To demonstrate transmission, sentinels were monitored for start and continuation of respiratory shedding. Viral load in oropharyngeal swabs of sentinels was measured by gRNA and sgRNA; swabs were

bioRxiv preprint doi: https://doi.org/10.1101/2021.07.26.453518; this version posted July 26, 2021. The copyright holder for this preprint (which was not certified by peer review) is the author/funder. This article is a US Government work. It is not subject to copyright under 17 USC 105 and is also made available for use under a CC0 license.

collected at 24, 48 and 72 hours post exposure to the donors. Exposure at 200 cm distance. Truncated violin plots depicting median, quantiles and individuals, blue = lineage A, red = B.1.1.7. Abbreviations: A, lineage A variant; g, genomic; sg, subgenomic.

bioRxiv preprint doi: https://doi.org/10.1101/2021.07.26.453518; this version posted July 26, 2021. The copyright holder for this preprint (which was not certified by peer review) is the author/funder. This article is a US Government work. It is not subject to copyright under 17 USC 105 and is also made available for use under a CC0 license.

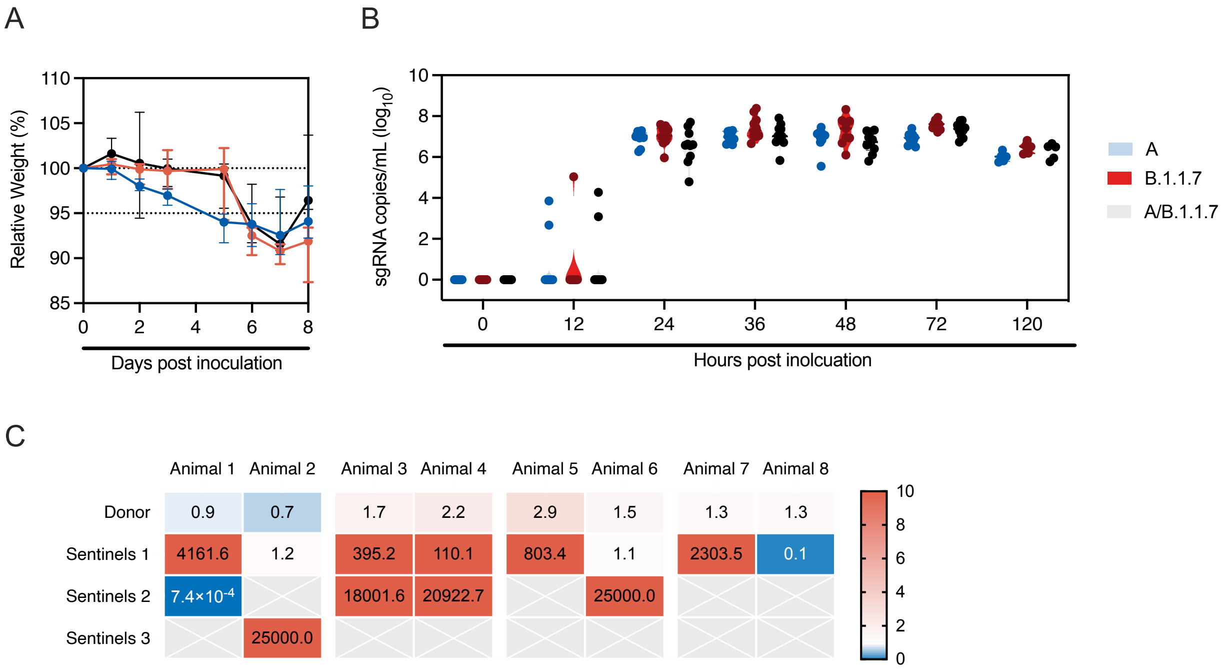

Supplementary Figure 3: Dual infection with lineage A and B.1.1.7 variant in the Syrian hamster. Animals (N = 10) were inoculated with both lineage A and B.1.1.7 variant with 10^2 $TCID_{50}$ via the intranasal route (1:1 ratio), **A.** Relative weight loss in hamsters after dual inoculation in comparison to lineage A or B.1.1.7 variant inoculation. Graph shows median and 95% CI, N = 10. **B.** Respiratory shedding as measured by sgRNA in oropharyngeal swabs collected at 0, 12, 24, 36, 48, 72 and 120 hours post inoculation. Truncated violin plots depicting median, quantiles and individual, N = 10. **C.** Donor animals (N = 8) were inoculated with both lineage A and B.1.1.7 variant with 10^2 $TCID_{50}$ via the intranasal route (1:1 ratio), and three groups of sentinels (Sentinels 1, 2 and 3) were exposed subsequently at 16.5 cm distance. Ratio of B.1.1.7 and lineage A variant found in oropharyngeal swabs taken at day 2 post exposure/inoculation for each individual donor and sentinel, measured by duplex-qRT-PCR and depicted by ct foldchange (B.1.1.7 over lineage A variant). Colors refer to scale on the right. Samples

bioRxiv preprint doi: https://doi.org/10.1101/2021.07.26.453518; this version posted July 26, 2021. The copyright holder for this preprint (which was not certified by peer review) is the author/funder. This article is a US Government work. It is not subject to copyright under 17 USC 105 and is also made available for use under a CC0 license.

for which only one variant was detected by PCR were set to 25,000. Abbreviations: A, lineage A variant; sg, subgenomic.

bioRxiv preprint doi: https://doi.org/10.1101/2021.07.26.453518; this version posted July 26, 2021. The copyright holder for this preprint (which was not certified by peer review) is the author/funder. This article is a US Government work. It is not subject to copyright under 17 USC 105 and is also made available for use under a CC0 license.

Supplementary Table 1: Aerosol Transmission Cage Validation Parameters. Special transmission cages were designed to model airborne transmission between Syrian hamsters. Volume of air (cages plus connection tube), air flow velocity in the tube and time for particles to traverse is provided at a cage air change rate of 30/h.

Cage system	Volume (L)	Linear velocity (cm/min)	Time (sec)
**Connecting tube (200 cm)**	1073	420	26.7692784
**Connecting tube (106 cm)**	945	370	15.059599
**Connecting tube (16.5 cm)**	837	327	2.80215197

bioRxiv preprint doi: https://doi.org/10.1101/2021.07.26.453518; this version posted July 26, 2021. The copyright holder for this preprint (which was not certified by peer review) is the author/funder. This article is a US Government work. It is not subject to copyright under 17 USC 105 and is also made available for use under a CC0 license.

Supplementary Table 2: anti-spike ELISA results for sentinels in aerosol transmission studies. Presence of SARS-CoV-2 spike IgG antibodies in sentinels co-housed at 16.5, 106 and 200 cm distance from lineage A or B.1.1.7(*) variant inoculated donor hamsters. Detected in serum obtained 14 days post exposure. negative (neg): optical density (at 450 nm) < 0.124, positive (pos) optical density (at 450 nm) ≥ 0.124.

Condition	Animal	Seroconverted
**16.5 cm**	S1	pos
	S2	pos
	S3	pos
	S4	pos
**106 cm**	S1	pos*
	S2	pos*
	S3	pos*
	S4	pos*
	S5	pos
	S6	pos
	S7	pos
	S8	pos
**200 cm**	S1	pos*
	S2	pos*
	S3	pos*
	S4	pos*
	S5	pos
	S6	pos
	S7	pos
	S8	pos

bioRxiv preprint doi: https://doi.org/10.1101/2021.07.26.453518; this version posted July 26, 2021. The copyright holder for this preprint (which was not certified by peer review) is the author/funder. This article is a US Government work. It is not subject to copyright under 17 USC 105 and is also made available for use under a CC0 license.

Supplementary Table 3: Pathological assessment of lungs collected at 5 days post exposure. Donor animals (N = 8) were inoculated with both lineage A and B.1.1.7 variant with 10^2 $TCID_{50}$ via the intranasal route (1:1 ratio), and three groups of sentinels (Sentinels 1, 2 and 3) were exposed subsequently at 16.5 cm distance. At five days post exposure, lungs were observed for gross pathology.

Identification	COVID-19 phenotype	Gross pathological assessment
**Sentinel 1.1**	N/A	bilateral focally extensive dorsal poorly circumscribed dark red discoloration
**Sentinel 1.2**	N/A	multifocal poorly circumscribed dark red discoloration
**Sentinel 1.3**	Yes	multifocal to coalescing foci of well circumscribed dark red discoloration suggestive of interstitial pneumonia
**Sentinel 1.4**	Yes	multifocal foci of well circumscribed dark red discoloration suggestive of interstitial pneumonia
**Sentinel 1.5**	N/A	focus of discoloration on the ventral margin on the left lobe, but no clear foci
**Sentinel 1.6**	Yes	multifocal foci of well circumscribed dark red discoloration suggestive of interstitial pneumonia
**Sentinel 1.7**	Yes	multifocal to coalescing foci of well circumscribed dark red discoloration suggestive of interstitial pneumonia
**Sentinel 1.8**	Yes	multifocal foci of well circumscribed dark red discoloration suggestive of interstitial pneumonia
**Sentinel 2.1**	N/A	multifocal foci of poorly circumscribed dark red discoloration suggestive of interstitial pneumonia
**Sentinel 2.2**	N/A	focally extensive foci of poorly circumscribed dark red discoloration
**Sentinel 2.3**	No	normal
**Sentinel 2.4**	No	normal
**Sentinel 2.5**	No	normal
**Sentinel 2.6**	No	normal
**Sentinel 2.7**	N/A	focally extensive foci of poorly circumscribed dark red discoloration
**Sentinel 2.8**	N/A	focally extensive foci of poorly circumscribed dark red discoloration
**Sentinel 3.1**	No	normal
**Sentinel 3.2**	No	normal
**Sentinel 3.3**	No	normal
**Sentinel 3.4**	No	normal
**Sentinel 3.5**	No	normal
**Sentinel 3.6**	No	normal
**Sentinel 3.7**	No	normal
**Sentinel 3.8**	No	normal

bioRxiv preprint doi: https://doi.org/10.1101/2021.07.26.453518; this version posted July 26, 2021. The copyright holder for this preprint (which was not certified by peer review) is the author/funder. This article is a US Government work. It is not subject to copyright under 17 USC 105 and is also made available for use under a CC0 license.

Supplementary Table 4: qRT-PCR and sequencing results for donor and sentinel animals. Donor animals (N = 8) were inoculated with both lineage A and B.1.1.7 variant with 10^2 $TCID_{50}$ via the intranasal route (1:1 ratio), and three groups of sentinels (Sentinels 1, 2 and 3) were exposed subsequently at 16.5 cm distance. Viral load in copies/reaction (measured by qRT-PCR) and percentage of B.1.1.7 detected in oropharyngeal swabs taken at day 2 post exposure for each individual donor and sentinel, determined by deep sequencing (expressed as %) and expressed as fold-change over lineage A as measured by duplex-qRT-PCR.

Animal	raw reads	qc reads	Viral RNA copies/reaction	% B.1.1.7	PCR fold-change
**Donor 1**	9697	9289	1239	54	0.87408891
**Donor 2**	77807	75255	85054	45	0.67126948
**Donor 3**	78379	75218	103141	67.66667	1.69959284
**Donor 4**	87992	85444	101401	70	2.1594464
**Donor 5**	85062	82728	199462	74.66667	2.93509401
**Donor 6**	135883	132657	254909	62	1.48700317
**Donor 7**	45156	43638	71343	57.66667	1.2711726
**Donor 8**	93687	91017	93356	57.66667	1.30263123
**Sentinel 1.1**	65408	63416	126315	99	4161.61905
**Sentinel 1.2**	41250	39943	38272	47	1.18602528
**Sentinel 1.3**	112221	107500	20842	98.33333	395.151923
**Sentinel 1.4**	107062	101891	62637	96	110.145084
**Sentinel 1.5**	91154	88209	42500	96.66667	803.405938
**Sentinel 1.6**	136060	131881	16994	58	1.12334412
**Sentinel 1.7**	44594	43114	115352	98	2303.5488
**Sentinel 1.8**	99311	96261	169096	16	0.12975316
**Sentinel 2.1**	131545	126930	29513	failed	0.00073727
**Sentinel 2.3**	33284	31942	4003	100	18001.5885
**Sentinel 2.4**	25906	25000	4056	100	20922.7397
**Sentinel 2.6**	10848	10219	522	failed	* only B.1.1.7 detected
**Sentinel 3.2**	154787	141295	65	failed	* only B.1.1.7 detected

bioRxiv preprint doi: https://doi.org/10.1101/2021.07.26.453518; this version posted July 26, 2021. The copyright holder for this preprint (which was not certified by peer review) is the author/funder. This article is a US Government work. It is not subject to copyright under 17 USC 105 and is also made available for use under a CC0 license.

Supplementary Table 5: Duplex-qRT-PCR Primers and Probes

Primer/probe	Sequence (5'→ 3')
**VM3256-RML-(drop out)**	VIC-TGTTACTTGGTTCCATGCTATACATG-ZEN-IBHQ
**VM3256-RML-(detection)**	FAM-GTTCCATGCTATCTCTGGGACC--ZEN-IBHQ
**VM3254-RML-F**	AAAGTTTTCAGATCCTCAG
**VM3255-RML-R**	GTTAGACTTCTCAGTGGAAG

bioRxiv preprint doi: https://doi.org/10.1101/2021.07.26.453518; this version posted July 26, 2021. The copyright holder for this preprint (which was not certified by peer review) is the author/funder. This article is a US Government work. It is not subject to copyright under 17 USC 105 and is also made available for use under a CC0 license.

Supplementary Table 6: Sequence results of virus stock B.1.1.7

ORF	a.a. change	Percentage
nsp6	D165G	14
nsp6	L257F	18
nsp7	V11I	13
